# The Predictors of Conduction Disturbances Following Transcatheter Aortic Valve Replacement in Patients With Bicuspid Aortic Valve: A Multicenter Study

**DOI:** 10.3389/fcvm.2021.757190

**Published:** 2021-11-29

**Authors:** Yuchao Guo, Dao Zhou, Mengqiu Dang, Yuxing He, Shenwei Zhang, Jun Fang, Shili Wu, Qiong Huang, Lianglong Chen, Yiqiang Yuan, Jiaqi Fan, Hasan Jilaihawi, Xianbao Liu, Jian'an Wang

**Affiliations:** ^1^Department of Cardiology, Second Affiliated Hospital, Zhejiang University School of Medicine, Hangzhou, China; ^2^Department of Cardiology, Zhengzhou Cardiovascular Hospital (The Seventh People' Hospital of Zheng Zhou), Zhengzhou, China; ^3^Department of Cardiology, Fujian Heart Medical Center, Fujian Institute of Coronary Heart Disease, Fujian Medical University Union Hospital, Fuzhou, China; ^4^Department of Cardiology, The First Affiliated Hospital of Bengbu Medical College, Bengbu, China; ^5^Department of Cardiology, Henan Provincial Chest Hospital, Zhengzhou, China; ^6^Heart Valve Center, NYU Langone Health, New York City, NY, United States; ^7^Department of Cardiology, Second Affiliated Hospital, Zhejiang University School of Medicine, Hangzhou, China

**Keywords:** bicuspid aortic stenosis, conduction disturbances, TAVR–transcatheter aortic valve replacement, membranous septum, oversizing ratio

## Abstract

**Objective:** To evaluate the predictors of new-onset conduction disturbances in bicuspid aortic valve patients using self-expanding valve and identify modifiable technical factors.

**Background:** New-onset conduction disturbances (NOCDs), including complete left bundle branch block and high-grade atrioventricular block, remain the most common complication after transcatheter aortic valve replacement (TAVR).

**Methods:** A total of 209 consecutive bicuspid patients who underwent self-expanding TAVR in 5 centers in China were enrolled from February 2016 to September 2020. The optimal cut-offs in this study were generated from receiver operator characteristic curve analyses. The infra-annular and coronal membranous septum (MS) length was measured in preoperative computed tomography. MSID was calculated by subtracting implantation depth measure on postoperative computed tomography from infra-annular MS or coronal MS length.

**Results:** Forty-two (20.1%) patients developed complete left bundle branch block and 21 (10.0%) patients developed high-grade atrioventricular block after TAVR, while 61 (29.2%) patients developed NOCDs. Coronal MS <4.9 mm (OR: 3.08, 95% CI: 1.63–5.82, *p* = 0.001) or infra-annular MS <3.7 mm (OR: 2.18, 95% CI: 1.04–4.56, *p* = 0.038) and left ventricular outflow tract perimeter <66.8 mm (OR: 4.95 95% CI: 1.59–15.45, *p* = 0.006) were powerful predictors of NOCDs. The multivariate model including age >73 years (OR: 2.26, 95% CI: 1.17–4.36, *p* = 0.015), Δcoronal MSID <1.8 mm (OR: 7.87, 95% CI: 2.84–21.77, *p* < 0.001) and prosthesis oversizing ratio on left ventricular outflow tract >3.2% (OR: 3.42, 95% CI: 1.74–6.72, *p* < 0.001) showed best predictive value of NOCDs, with c-statistic = 0.768 (95% CI: 0.699–0.837, *p* < 0.001). The incidence of NOCDs was much lower (7.5 vs. 55.2%, *p* < 0.001) in patients without Δcoronal MSID <1.8 mm and prosthesis oversizing ratio on left ventricular outflow tract >3.2% compared with patients who had these two risk factors.

**Conclusion:** The risk of NOCDs in bicuspid aortic stenosis patients could be evaluated based on MS length and prosthesis oversizing ratio. Implantation depth guided by MS length and reducing the oversizing ratio might be a feasible strategy for heavily calcified bicuspid patients with short MS.

## Introduction

New-onset conduction disturbances (NOCDs) such as complete left bundle branch block and high-grade atrioventricular block are common complications after transcatheter aortic valve replacement (TAVR), which may result in permanent pacemaker implantation (PPMI). Despite rapid advances in procedure techniques and new generation prosthesis, the rates of new-onset complete left bundle branch block (10.5–52.3%) and PPMI (2.3–36.1%) after TAVR remain high, especially in TAVR with self-expanding valve (1–4). NOCDs and PPMI were previously believed to mainly impair mid-term improvement of left ventricular remodeling or left ventricular ejection fraction after TAVR (4, 5). However, a recent pooled analysis suggested that new-onset persistent left bundle branch block and permanent pacemaker implantation were associated with the increased risks of 1-year heart-failure rehospitalization and all-cause mortality (3).

The pre-procedural NOCDs risk assessment before TAVR is crucial for procedural planning both for elder patients prone to conduction disturbances or younger recipients with long life expectancy. Baseline conduction disturbances, such as pre-existing right bundle branch block and left bundle branch block, are traditional predictors of NOCDs (2). More recently, studies have suggested that anatomy and procedural factors regarding membranous septum length (MS), device landing zone calcification, and implantation depth are associated with NOCDs. In a recent study, Jilaihawi et al. (6) provided a useful prediction model and procedural strategy to minimize PPMI in patients with tricuspid aortic valve who underwent self-expanding TAVR. Nevertheless, data on predictors and strategies to reduce NOCDs in severe aortic stenosis patients with bicuspid aortic valve (BAV) are limited (7). A recent propensity-matched study from Hamdan et al. (7) reported that MS length was shorter in BAV patients and associated with increased risk of conduction disturbances. On the other hand, over the past few years, undersizing of prosthesis especially in highly calcified bicuspid patients has been a topic. Several supra-annular sizing methods have been raised to select a smaller prosthesis with few paravalvular leakage and high device success (8–10), which might theoretically lower the incidence of conduction disturbances. Consequently, we performed this study to evaluate predictors of NOCDs in BAV patients using self-expanding valve.

## Materials and Methods

### Study Population and Procedure

**Figure 1:**
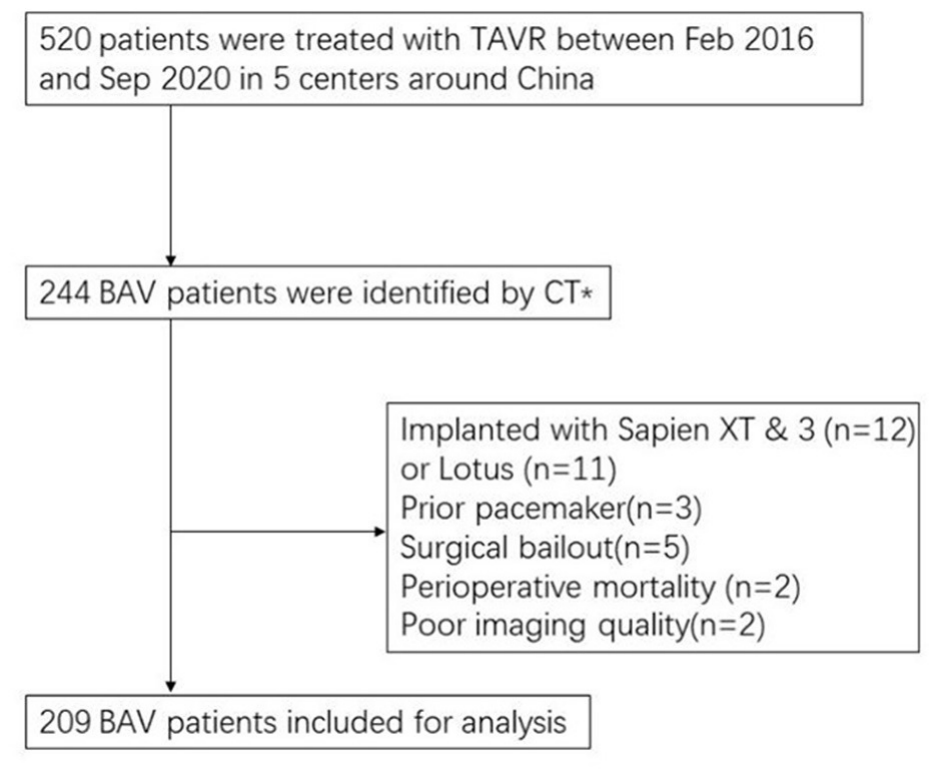
Flow chart of study population. TAVR, Transcatheter aortic valve replacement. *CT classification: bicuspid patients were classified into “bicommissural non-raphe-type,” “bicommissural raphe-type,” and “tricommissural raphe-type BAV” according to pre-procedural multislice computed tomography.

A total of 520 consecutive patients who underwent TAVR for severe aortic stenosis in 5 centers in China were retrospectively included from February 2016 to September 2020. Two hundred forty-four BAV patients were identified by two experienced cardiologists (YH and QZ) and were confirmed by two authors (YG and DZ) following Jilaihawi's classification (11, 12). In this study, the term “Type 0” was equivalent to “bicommissural non-raphe-type,” and “Type 1” was considered same as “bicommissural raphe-type,” while the term “Tricommissural BAV (T-BAV)” was equally used to describe Tricommissural raphe-type BAV (11). After excluding 35 patients based on the following exclusion criteria: 1) with prior pacemaker implantation (*n* = 3); 2) needed urgent transfer to open surgery (*n* = 5); 3) with poor pre-operative CT imaging quality (*n* = 2); 4) using balloon-expandable valve or mechanically-expandable valve (*n* = 23); 5) suffering perioperative death (*n* = 1), a total of 209 BAV patients were included in our study (Figure 1). Written informed consent was obtained from each participant. The study was approved by the local ethics committee and complied with the Declaration of Helsinki.

In our study, pre-operative electrocardiography was performed in all patients. Holter monitoring was performed in high risk patients to identify potential pre-operative cardiac arrhythmia. The decision to perform TAVR was made by a multidisciplinary heart team. Most TAVR procedures were completed through transfemoral access under general anesthesia. self-expanding valves including Venus A (Venus Medtech, Hangzhou, China), Vitaflow (Microport, Shanghai, China), TaurusOne (Peijia Medical, Suzhou, China), and their series were used in this study. The selection of valve size was made by the heart team based on preoperative cardiac computed tomography (CT) analysis and the fluoroscopy during balloon valvuloplasty. A modified Supra-annular structure assessment method by balloon sizing was recommended for all operators in this study (8). Patients underwent balloon valvuloplasty with a Z-Med balloon (NuMED, Hopkinton, NY). The Z-med balloon size was determined based on the lowest range of annulus perimeter driven diameter. For example, a 20-mm Z-Med balloon was used in annulus perimeter driven diameter range of 20–23 mm. Smaller balloon size was recommended in case of potential risk of annular rupture. If waist sign on the balloon and less than mild regurgitation were simultaneously observed with a contrast injection, a smaller prosthesis other than manufacturer recommendation was chosen based on the balloon size.

Post-procedural electrocardiogram monitoring or remote monitoring was routinely used. Echocardiography and electrocardiography were performed before discharge and at 1 months' follow-up. Also, cardiac contrast-enhanced electrocardiography-gated CT was performed before discharge or at 1-month examination in most patients. Left bundle branch block and high-grade atrioventricular block in our study were defined as reported in a previous study (13). Patients with NOCDs were defined as patients with new-onset persistent complete left bundle branch block or with high-grade atrioventricular block before discharge.

### Image Aquasition and Analysis

Cardiac contrast-enhanced electrocardiography-gated multidetector computed tomography (MDCT) was performed on PHILIPS Brilliance iCT 256 or GE Revolution CT using collimation of 0.6 or 0.8 mm, 100 or 120 kV. Fluoroscopy was recorded with a classic coplanar view after valve final deployment to assess final prosthesis depth at NCC (non-coronary cusp). CT or fluoroscopy imaging were analyzed by two authors (YG and DZ) applying a single-blind method, with CT's measurement on 3 mensio Valves software version 9.1/10.0 (Bilthoven, the Netherlands) and fluoroscopy on RadiAnt DICOM Viewer Software version 2020.1 (Medixant, Poznan, Poland). A tertiary researcher (YH) analyzed the imaging separately in the situation of great difference on image analysis.

**Figure 2:**
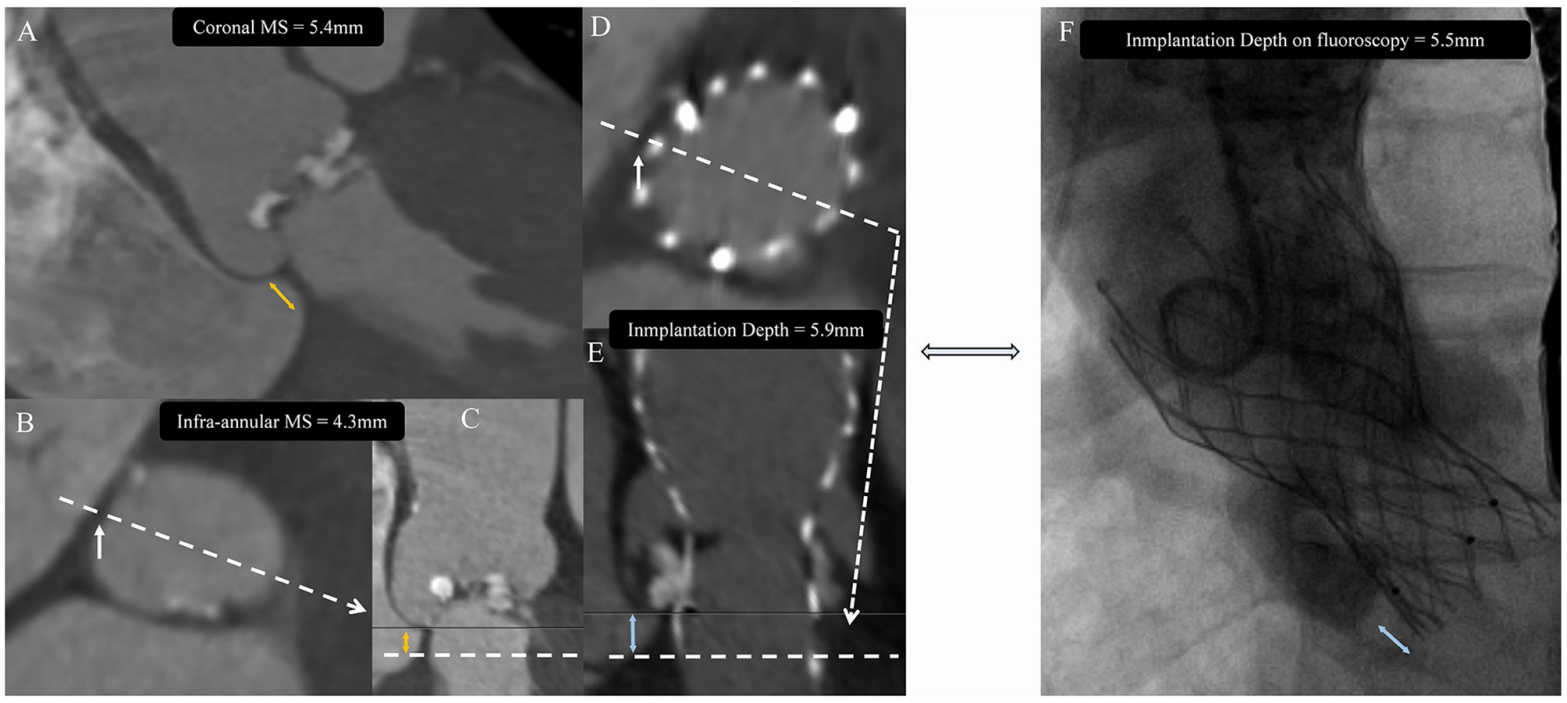
Image analysis protocol. The membranous septum (MS) was measured on coronal view **(A)** as coronal MS or on stretched vessel view **(C)** as infra-annular MS at the tricuspid insertion point **(B)**. Device's implantation depth (ID) was measured on post-operative CT **(D,E)** and was compared with the measurement on post-releasing fluoroscopy **(F)**. Yellow double arrow indicates membranous septum length (MS). White arrow indicates tricuspid insertion point. Blue double arrow indicates prosthesis implantation depth (ID).

The length of infra-annular MS was measured as the distance from the annulus to the vertex of the muscular ventricular septum on stretch vessel imaging close to the tricuspid valve insertion point. Coronal MS lengths were measured in the coronal view, as previously described (6, 14). Device's implantation depth (ID) was measured on post-operative CT from the plane where the prosthesis metal stent disappeared (in line with the MS) to the annulus. Implantation depth measured on post-release fluoroscopy was also evaluated on NCC direction (6, 15) (Figure 2). The ΔMSID or Δcoronal MSID was calculated by subtracting implantation depth from infra-annular MS or coronal MS length. The severity of valve calcification was classified as grade 1 to 4, and the calcification of LVOT plane was described in a qualitative fashion and graded as none, mild, moderate, or severe, as described in previous studies (16, 17). The oversizing ratio was calculated using device geometrical data from manufacturers by the following formulas: *oversizing by area (%)* = *(prosthesis inflow nominal area/measured area* − *1)* × *100%*, and *oversizing by perimeter (%)* = *(prosthesis inflow nominal perimeter/measured perimeter* − *1)* × *100%* (18–20).

### Statistical Analysis

Category variables were presented as numbers (%) and were tested by Chi-square or Fisher exact test. Continuous variables were expressed as mean ± standard deviation or median [interquartile range (IQR)] and were compared with Student's *t*-test or Mann Whitney *U*-test based on distribution type tested by Shapiro-Wilk test. Correlation analysis was conducted on prosthesis depth measured on CT and fluoroscopy, coronal MS and infra-annular MS, as well as ΔMSID and coronal ΔMSID using Spearman correlation test. A 2-tailed *P* < 0.05 was considered as a significant difference. The optimal cut-off values of continuous variables were determined by receiver operating characteristic (ROC) analysis. The variables with a *P* < 0.05 in univariate regression analyses were entered into multivariate logistic regression models with forward likelihood ratio method, which contained pre-operative variables or included both pre- and post-operative variables. All statistical analyses were performed using SPSS software version 20.0 (IBM, Armonk, New York).

## Results

### Patients' Baseline Characteristics

Baseline clinical characteristics and CT measurement results are shown in Tables 1, 2. The mean age of the population was 75.12 ± 6.79, and the median Society of Thoracic Surgeons (STS) score was 5.487 (3.626–9.052). Among 209 patients, 99 (47.4%) had type 0, 79 (37.8%) had type 1 and 31 (14.8%) had tricommissural BAV. Most baseline characteristics were similar between three different types of BAV except for some differences in anatomy measurement (Supplementary Tables 1A–C).

**Table 1:** Baseline clinical characteristics of bicuspid aortic stenosis patients and NOCDs.

	**Total (*n* = 209)**	**No NOCDs (*n* = 148)**	**NOCDs (*n* = 61)**	***p*-value**
**Baseline clinical variables**				
Age, yrs	75.12 ± 6.79	74.41 ± 6.83	76.85 ± 6.41	**0.017**
Male	128 (61.2%)	96 (64.9%)	32 (52.5%)	0.094
Body mass index, kg/m^∧^2	22.53 ± 3.11	22.66 ± 3.19	22.23 ± 2.89	0.364
Diabetes mellitus	41 (19.6%)	24 (16.2%)	17 (27.9%)	0.054
Hypertension	100 (47.8%)	68 (45.9%)	32 (52.5%)	0.391
Chronic obstructive pulmonary disease	43 (20.6%)	30 (20.3%)	13 (21.3%)	0.866
Chronic kidney disease stage 4–5	4 (1.9%)	4 (2.7%)	0 (0%)	0.324
NYHA classification				0.562
II	24 (11.5%)	15 (10.1%)	9 (14.8%)	
III	105 (50.2%)	77 (52%)	28 (45.9%)	
IV	80 (38.3%)	56 (37.8%)	24 (39.3%)	
STS score, %	5.487 (3.626–9.052)	5.485 (3.697–9.295)	5.487 (3.425–8.882)	0.632
**Baseline electrocardiographic variables**				
Atrial fibrillation/flutter	32 (15.3%)	22 (14.9%)	10 (16.4%)	0.780
Pre-existing LBBB	18 (8.6%)	18 (12.2%)	0 (0%)	–
Pre-existing RBBB	17 (8.1%)	9 (6.1%)	8 (13.1%)	0.101
**Baseline echocardiographic variables**				
Mean gradient, mmHg	56.0 (43.0–70.5)	56.5 (43.0–72.5)	53.0 (42.0–70.0)	0.428
Max velocity, m/s	4.90 (4.25–5.52)	4.89 (4.24–5.42)	4.90 (4.25–5.53)	0.970
Aortic regurgitation grade				0.747
None	43 (20.6%)	30 (20.3%)	13 (21.3%)	
Mild	104 (49.8%)	71 (48.0%)	33 (54.1%)	
Moderate	43 (20.6%)	32 (21.6%)	11 (18%)	
Severe	19 (9.1%)	15 (10.1%)	4 (6.6%)	
LVEF, %	57.0 (46.0–63.4)	55.9 (42.3–63.0)	58.8 (50.9–64.5)	0.127

*Values are presented as mean ± SD or median (Quartile1–Quartile3) or n (%). p-values in bold are statistically significant*.

*NOCDs, new-onset conduction disturbances; NYHA, New York heart association; LBBB, left bundle branch block; RBBB, right bundle branch block; STS, society of thoracic surgeons; LVEF, left ventricular ejection fraction*.

**Table 2:** Computed tomography characteristics of bicuspid aortic stenosis patients and NOCDs.

	**Total (*n* = 209)**	**No NOCDs (*n* = 148)**	**NOCDs (*n* = 61)**	***p*-value**
BAV classification				0.652
Type 0	99 (47.4%)	70 (47.3%)	29 (47.5%)	
Type 1	79 (37.8%)	58 (39.2%)	21 (34.4%)	
T-BAV	31 (14.8%)	20 (13.5%)	11 (18.0%)	
Valve calcification grade, class III or IV	177 (84.7%)	127 (85.8%)	50 (82.0%)	0.483
Annulus area, mm^∧^2	457.3 (408.3–525.8)	466 (405.4–549.4)	440.5 (408.7–509.4)	0.077
Annulus area derived diameter, mm	24.1 (22.8–25.9)	24.4 (22.7–26.5)	23.7 (22.9–25.5)	0.082
Annulus perimeter, mm	77.2 (73.2–82.9)	77.6 (73.2–84.5)	75.7 (73.2–81.2)	0.072
Annulus perimeter derived diameter, mm	24.6 (23.3–26.4)	24.7 (23.3–26.9)	24.1 (23.3–25.8)	0.065
Annular eccentricity index	0.23 ± 0.07	0.23 ± 0.07	0.23 ± 0.07	0.819
LVOT area, mm^∧^2	488.9 (400.2–602.8)	496.6 (412.6–611.4)	461.5 (389.8–564.7)	**0.041**
LVOT area derived diameter, mm	25.0 (22.6–27.7)	25.2 (22.9–27.9)	24.2 (22.3–26.9)	**0.041**
LVOT perimeter, mm	83.8 (75.4–92.7)	84.6 (76.5–94.1)	80.9 (73.9–88.3)	**0.032**
LVOT perimeter derived diameter, mm	26.4 (23.9–29.1)	26.8 (23.9–29.3)	25.1 (23.7–28.5)	0.058
LVOT eccentricity index	0.31 ± 0.08	0.31 ± 0.09	0.32 ± 0.08	0.372
LVOT calcification	36 (17.2%)	30 (20.3%)	6 (9.8%)	0.069
LVOT/annulus perimeter ratio	1.06 ± 0.17	1.07 ± 0.17	1.03 ± 0.17	0.139
LVOT/annulus area ratio	1.04 ± 0.10	1.05 ± 0.10	1.03 ± 0.10	0.316
SOV mean diameter, mm	32.97 ± 3.53	33.15 ± 3.63	32.55 ± 3.25	0.268
STJ average diameter, mm	31.4 (29.0–34.4)	31.6 (29.0–34.8)	30.7 (28.7–33.5)	0.287
STJ height, mm	22.6 (20.0–26.1)	22.8 (20.4–26.3)	21.6 (19.5–25.0)	0.115
Ascending aorta diameter, at 40 mm	38.85 ± 3.88	39.06 ± 3.83	38.33 ± 4.00	0.221
Ascending aorta diameter, Max	42.62 ± 4.68	43.02 ± 4.68	41.63 ± 4.55	**0.050**
RCA height, mm	16.9 (14.9–19.4)	17.2 (15.1–19.8)	16.3 (14.1–19.0)	0.069
LCA height, mm	15.4 (13.3–18.4)	15.7 (13.2–18.3)	14.9 (13.3–18.9)	0.717
Aortic root angulation	52.82 ± 10.49	53.18 ± 9.96	51.93 ± 11.71	0.435
Infra-annular MS length, mm	2.3 (1.2–3.9)	2.3 (1.0–4.1)	2.3 (1.5–3.4)	0.747
Infra-annular MS length <3.7 mm	150 (71.8%)	100 (67.6%)	50 (82.0%)	**0.036**
Coronal MS length, mm	5.7 (4.7–7.0)	6.0 (5.0–7.2)	5.1 (4.1–6.3)	**<0.001**
Coronal MS length <4.9 mm	60 (28.7%)	32 (21.6%)	28 (45.9%)	**<0.001**

*Values are presented as mean ± SD or median (Quartile1–Quartile3) or n (%). p-values in bold are statistically significant. BAV, bicuspid aortic valve; T-BAV, tricommissural bicuspid aortic valve; LVOT, left ventricular outflow tract; SOV, sinus of Valsalva; STJ, sinotubular junction; LCA, left coronary height; RCA, right coronary artery; MS, membranous septum; other abbreviations as in Table 1*.

### Procedural Characteristics and Relevant Outcomes

Most patients (205, 98.1%) underwent TAVR through transfemoral access, and the remaining 4 patients through transcarotid access. First-generation self-expanding valves were used in 174 (83.3%) patients, while next-generation valve with recapturable features was used in 35(16.7%) patients. One hundred fifty-eight (75.6%) patients received undersized prosthesis based on supra-annular balloon sizing. A total of 204 (97.6%) patients received pre-dilatation and 150 (71.8%) patients underwent post-dilatation. The overall pre-discharge mortality rate was 0.5%; in-hospital stroke rate was 1%, and the rate of second prosthesis implantation was 8.1%. Forty-two (20.1%) patients developed complete left bundle branch block and 21 (10.0%) patients developed high-grade atrioventricular block after TAVR, while 61 (29.2%) patients developed NOCDs. Sixteen (7.7%) patients received pacemaker implantation during the hospital stay, and 1 patient needed pacemaker implantation for high-grade atrioventricular block after discharge (10 days after discharge). The detailed pre- and post- operative arrhythmic characteristics of patients with new-onset high-grade atrioventricular block are described in Supplementary Table 2. In a single center analysis of 161 patients recruited in Second Affiliated Hospital Zhejiang University School of Medicine. The VARC-2 device success rate (absence of procedural mortality AND correct positioning of a single valve AND mean gradient <20 mmHg or peak velocity <3 m/s, AND no moderate or severe regurgitation) was 84.5%, with 13 (8.1%) cases of moderate PVL. Mean post-procedural gradient was 12.5 ± 6.8 mmHg.

### MS Length: Reproducibility

The reproducibility of coronal MS and infra-annular MS length measurement was assessed by comparing repeated measures of 18 randomly selected consecutive cases, which were performed by two experienced observers (YG and DZ). The paired samples correlation coefficient of interobserver measurements of coronal MS and infra-annular MS length was 0.855 (*p* < 0.001), 0.976 (*p* < 0.001), respectively. The paired difference was 0.383 mm [95% confidence interval (CI): −0.050–0.818 mm, *p* = 0.080), 0.206 mm (95% CI: −0.012–0.423 mm, *P* = 0.063), respectively. For intraobserver measurements, the paired samples correlation coefficient was 0.883 (*p* < 0.001), 0.982 (*p* < 0.001) and the paired difference was 0.278 mm (95% CI: −0.109–0.665 mm, *p* = 0.148), −0.167 mm (95% CI: −0.356–0.022 mm, *p* = 0.082), respectively.

### Membranous Septum and Implantation Depth Measurement Results

The overall median coronal MS was 5.7 [Interquartile range (IQR): 4.7–7.0] mm and the median infra-annular MS was 2.3 (IQR: 1.2–3.9) mm. In the intergroup analysis, Type 0 BAV patients had a shorter coronal MS compared with Type 1 BAV and T-BAV (5.58 ± 1.92 vs. 6.31 ± 2.25 mm, *p* = 0.022; 5.58 ± 1.92 vs. 6.44 ± 2.04 mm, *p* = 0.046) while no difference could be found in infra-annular MS between three groups (Supplementary Table 1C). Besides, correlations between coronal MS and infra-annular MS were moderate (*R* = 0.515; *P* < 0.01, Supplementary Figure 1A). The mean implantation depth on fluoroscopy or CT was 6.84 ± 4.36 or 6.37 ± 4.11 mm, respectively. There was a significant positive correlation between implantation depth measured by fluoroscopy and by CT (*R* = 0.761; *P* < 0.01, Supplementary Figure 1B).

### Patients and Procedural Predictors of Conduction Disturbances and PPMI

**Figure 3:**
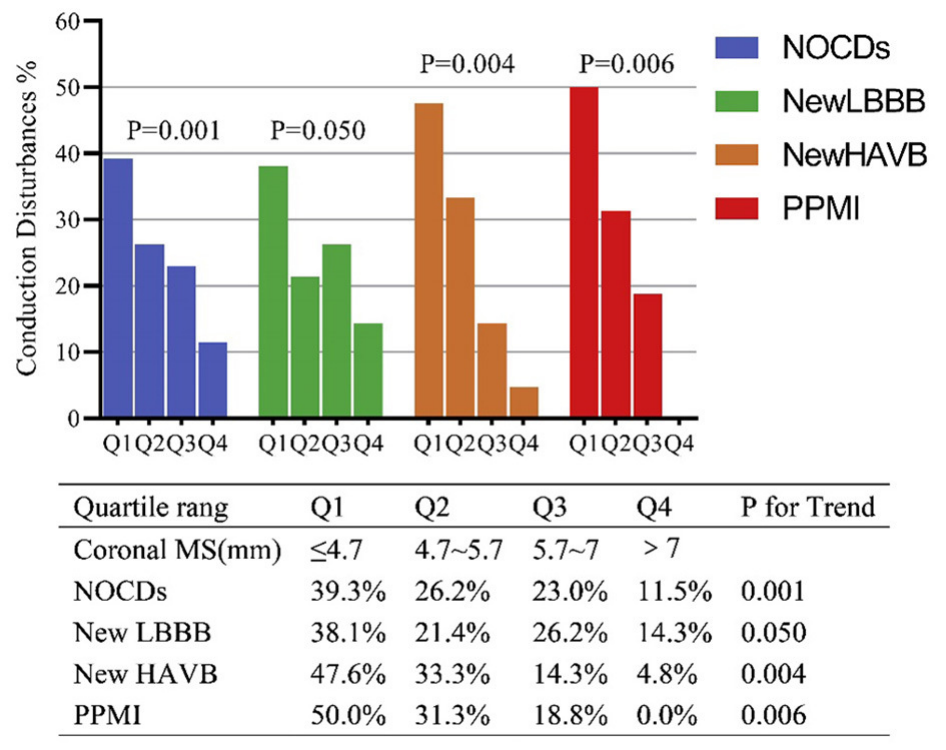
Incidence of NOCDs according to quartiles of coronal MS. MS, membranous septum; LBBB, left bundle branch block; HAVB, high-grade.

Baseline predictors of NOCDs were advanced age and smaller aortic root anatomy, including LVOT and ascending aorta (Tables 1, 2). Notably, patients who developed NOCDs had a significantly shorter coronal MS [5.1 (IQR: 4.1–6.3) mm vs. 6.0 (IQR: 5.0–7.2) mm, *p* < 0.001] compared with no NOCDs patients while no difference of infra-annular MS length could be found between two groups [2.3 (IQR: 1.5–3.4) mm vs. 2.3 (IQR: 1.0–4.1) mm, *p* = 0.747]. However, the proportion of infra-annular MS length <3.7 mm was higher in the NOCDs group (82.0 vs. 67.6%, *p* = 0.036) with the optimal cut-off determined by ROC curve. More patients with coronal MS length <4.9 mm could also be found in NOCDs groups (45.9 vs. 21.6%, *p* < 0.001). Besides, after dividing coronal MS into four quartiles, we found a significant inverse distribution of NOCDs between the four groups. Twenty-four (39.3%) out of 61 NOCDs and 8 (50.0%) out of 16 PPMI occurred in coronal ≤ 4.7 mm (less than the first quartile, Q1) while 7 (11.5%) NOCDs and 0 (0.0%) PPMI occurred in coronal MS >7 mm (more than the third quartiles) (Figure 3). When considering the procedural factors, we found that oversizing ratio by annulus or LVOT, implantation depth, ΔMSID, and Δcoronal MSID were predictors of NOCDs (Table 3).

**Table 3:** Procedural characteristics and conduction abnormalities.

	**Total (*n* = 209)**	**No NOCDs (*n* = 148)**	**NOCDs (*n* = 61)**	***p*-value**
Oversizing by annulus perimeter, %	4.9 ± 8.7	3.8 ± 9	7.7 ± 7.2	**0.003**
Oversizing by annulus area, %	15.8 ± 19.4	13.4 ± 20.1	21.6 ± 16.6	**0.005**
Oversizing by LVOT perimeter, %	−1.2 ± 12.9	−3.1 ± 12.5	3.2 ± 13.0	**0.001**
Oversizing by LVOT area, %	9.1 (−7.8, 29.6)	7.0 (−10.0,26.1)	18.6 (2.6, 37.3)	**0.002**
Pre-dilatation	204 (97.6%)	145 (98%)	59 (96.7%)	0.630
Post-dilatation	150 (71.8%)	106 (71.6%)	44 (72.1%)	0.941
Second valve implantation	17 (8.1%)	10 (6.8%)	7 (11.5%)	0.273
**Post-conduction disturbances**				
Post-new LBBB	42 (20.1%)	0 (0%)	42 (68.9%)	
Post-new RBBB	6 (2.9%)	5 (3.4%)	1 (1.6%)	
Post-new HAVB	21 (10.0%)	0 (0.0%)	21 (34.4%)	
Post-PPMI	16 (7.7%)	0 (0.0%)	16 (26.2%)	
Implant depth, mm	6.3 (3.9, 9.0)	5.4 (3.7, 8.8)	7.3 (5.1, 10.2)	**0.005**
ΔMSID, mm	−4.0 (−6.6, −1.3)	−3.0 (−6.5, −0.6)	−5.1 (−7.3, −3.1)	**0.006**
Implant depth > Infra-annular MS length	173 (82.8%)	116 (78.4%)	57 (93.4%)	**0.009**
Δcoronal MSID, mm	−0.86 ± 4.85	−0.17 ± 5.07	−2.56 ± 3.78	**<0.001**

*Values are presented as mean ± SD or median (Quartile1, Quartile3) or n (%). P-values in bold are statistically significant*.

*HAVB, high-grade atrioventricular block; PPMI, permanent pacemaker implantation; ΔMSID, infra-annular MS length minus implantation depth on CT; Δcoronal MSID, coronal MS length minus implantation depth on CT; other abbreviations as in Tables 1, 2*.

### Univariate and Multivariate Predictors of New-Onset Conduction Disturbances

**Figure 4:**
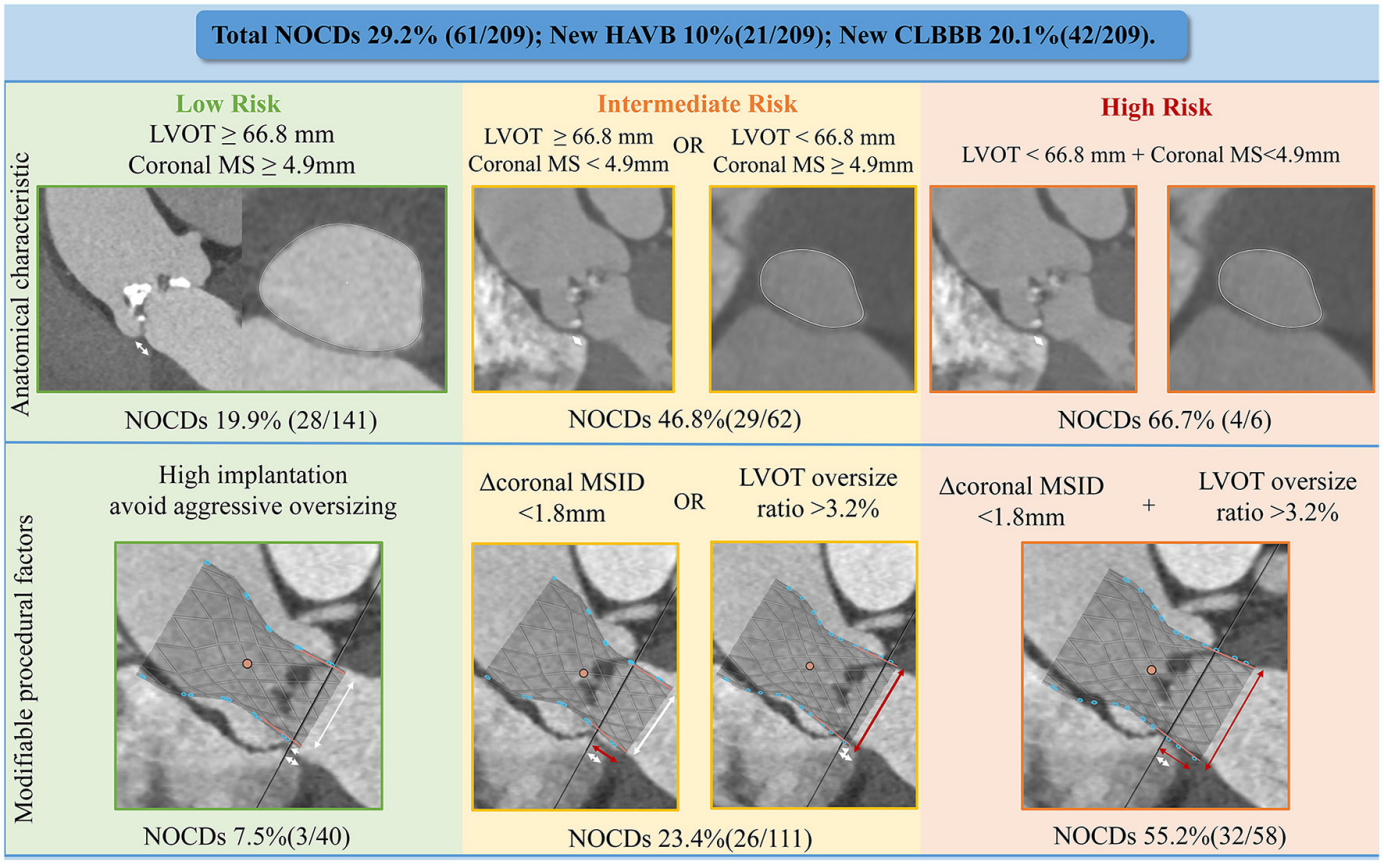
Predictive model of NOCDs. NOCDs, new-onset conduction disturbances; HAVB, high-grade atrioventricular block; CLBBB, complete left bundle branch block; LVOT, left ventricular outflow tract; Δcoronal MSID, coronal MS length minus implantation depth on CT.

Table 4 shows multivariate analysis results of predictors of NOCDs. The preprocedural multivariate logistic regression models revealed that age >73 years old, LVOT perimeter <66.8 mm, and Coronal MS <4.9 mm or infra-annular MS <3.7 mm were independent predictors of NOCDs. When taking post-procedural variables into consideration, the multivariate model including age, Δcoronal MSID, and oversizing by LVOT perimeter showed the best predictive value of NOCDs, with c-statistics = 0.768 (95% CI: 0.699–0.837, p <0.001). Besides, age > 73 years old, ΔMSID < -2.9 mm and oversizing by LVOT perimeter >3.2% were independent predictors of NOCDs in another model, which also had a good predictive value with c-statistics = 0.752 (95% CI: 0.679–0.824, *p* < 0.001). The detail comparison between predictive models was shown in Supplementary Table 3, which suggested a better predictive value of the model including MS measured on coronal view. According to our pre-operative multivariate model, patients could be classified as low, intermediate and high risk of NOCDs with the prevalence rates of 19.9, 46.8, 66.7% (Figure 4). When considering implantation depth and oversizing ratio by LVOT perimeter which could be mediated by operators, low, intermediate and high risk patients had rates of NOCDs of 7.5, 23.4, 55.2% (Figure 4).

**Table 4:** Multivariate logistic regression for predictors of new-onset conduction disturbances.

	**Multivariate analysis**					
	**Pre-procedural**				**Pre- and post-procedural**	
	***p*-value**	**OR (95% CI)**	***p*-value**	**OR (95% CI)**	***p-*value**	**OR (95% CI)**
Age > 73 yrs	0.024	2.18 (1.11–4.28)	0.019	2.29 (1.15–4.56)	0.002	3.07 (1.49–6.31)
LVOT perimeter <66.8 mm	0.013	4.39 (1.37–14.00)	0.019	4.09 (1.26–13.32)	–	–
Infra-annular MS <3.7 mm	0.040	2.22 (1.04–4.77)	–	–	–	–
Coronal MS <4.9 mm	–	–	0.001	3.14 (1.61–6.10)	–	–
Δcoronal MSID <1.8 mm	–	–	–	–	<0.001	7.87 (2.84–21.77)
Oversizing by LVOT perimeter >3.2%	–	–	–	–	<0.001	3.42 (1.74–6.72)

*Multivariate logistic regression included parameters with a p <0.05 without significant multicollinearity using forward Likelihood Ratio method*.

*OR, odds ratio; CI, confidence interval; other abbreviations as in Tables 1–3*.

### Subgroup Analysis of New High-Grade Atrioventricular Block or Implantation Depth Deeper Than MS Group

We also conducted a subgroup analysis focusing on new-onset high-grade atrioventricular block. The patients in new-onset high-grade atrioventricular block group had a higher rate of pre-existing right bundle branch block (33.3 vs. 5.3%, *p* < 0.001), shorter coronal MS [4.8 (IQR: 4.1–5.5) vs. 5.9 (IQR: 4.8–7.1), *p* = 0.003] and larger oversizing ratio by LVOT perimeter (4.59 ± 12.89 vs. −1.89 ± 12.82, *p* = 0.029) compared with the control group (Supplementary Table 4A). In univariate logistic regression analysis, coronal MS, pre-existing right bundle branch block, pre-dilatation and oversizing by LVOT perimeter were independent predictors of high-grade atrioventricular block, while pre-existing right bundle branch block (OR: 8.36, 95% CI: 2.50–27.89, *p* = 0.001), coronal MS <5.5 mm (OR:5.78, 95% CI: 1.75–19.12, *p* = 0.004) and oversizing ratio by LVOT perimeter >6.4% (OR: 3.80, 95% CI: 1.38–10.50, *P* = 0.010) remained powerful predictors in multivariate regression model with c-statistics = 0.805 (95% CI: 0.699–0.911, *p* < 0.001, Supplementary Table 4B).

In a subgroup analysis of patients with implantation depth larger than infra-annular MS, diabetes mellitus, older age, smaller aortic root morphology, and the larger oversizing ratio of prosthesis might contribute to new conduction disturbances (Supplementary Table 5A). We found that in this population, ΔMSID (OR: 0.97, 95% CI: 0.89–1.05, *p* = 0.968) was no longer an independent predictor. In multivariate logistic analysis, only age (OR: 1.07, 95% CI: 1.01–1.12, *p* = 0.020) and prosthesis oversizing ratio of LVOT perimeter (per 1%, OR: 1.05, 95% CI: 1.02–1.08, *p* = 0.001) remained strong predictors for new conduction disturbances (Supplementary Table 5B).

## Discussion

The main findings of the present study are following: 1) a model including age, LVOT perimeter, and coronal MS yielded best pre-procedural predictive value for NOCDs, while a model that included age, oversizing ratio by LVOT perimeter, and Δcoronal MSID had a best predictive value of NCODs; 2) the risk of NOCDs in BAV patients could be evaluated before TAVR procedure based on MS length and LVOT perimeter 3) MS length guide implantation with reduced size valve could be a feasible way to reduce the risk of NOCDs for BAV patients with short MS.

The clinical impact of PPMI and new-onset left bundle branch block after TAVR remains unclear. However, Faroux L's meta-analysis highlighted the adverse clinical impact of NOCDs (3). Recent data also indicated that the incidence of PPMI was still high and highly variable (21–24). The bicuspid aortic valve, previously thought of as a contraindication for TAVR owing to its anatomy, was gradually considered safe and feasible for TAVR (25–27). The rates of BAV in the normal population have been reported to be 0.5–2%, while BAV was quite common in patients who underwent surgical aortic valve replacement for aortic stenosis with the prevalence rate of almost 50% (28, 29). In our study, 244 out of 520 (46.9%) patients were consecutive BAV patients (6, 12) (Supplementary Tables 1A–C). Besides, BAV patients were often younger, which means they had more chance of suffering the adverse impact of NOCDs (30). Previous studies also suggested higher or similar risks of PPMI in BAV patients (31–33). In addition, self-expanding valves were widely used in clinical practice and were considered to have a significantly higher risk of PPMI than balloon-expandable valves (34). Nonetheless, the data presented here showed a postoperative new-onset high-grade atrioventricular block rate of 10.0% and a complete left bundle branch block rate of 20.1% in BAV patients, which suggested the acceptable NOCDs risks in our BAV populations. Accordingly, the present study aimed to identify predictors of NOCDs in BAV patients treated with SEV and potentially minimizing strategy. To the best of our knowledge, this is the first multicenter study that evaluated the predictors of NOCDs in a population with BAV using the self-expanding valve.

### The Membranous Septum and Implantation Depth

The relationship between NOCDs and anatomy factors, especially the membranous septum, has received increasing attention over recent years. It has been reported that the bundle was located at the edge of the membranous septum, then emerging as a left bundle branch near or beneath the LVOT (35). Different types of NOCDs occurred when corresponding bundle of his branches were oppressed and damaged by prosthesis metal stent or tissue edema (35).

The left bundle branch was vulnerable with a short MS. In our study, both infra-annular and coronal MS lengths were measured. The overall coronal MS 5.7 (IQR 4.7–7.0) mm and infra-annular MS 2.3(IQR: 1.2–3.9) mm were numerical shorter than previously reported tricuspid population, which was in accordance with Hamdan A's finding (6, 7, 36). The high predictive value of coronal MS suggested that clinicians should evaluate BAV patients' coronal MS length before TAVR procedure, which could be measured directly on Picture Archiving and Communication Systems. In addition to MS length, the distance between the membranous septum and implantation depth was a more important predictor of NOCDs. We found a Δcoronal MSID of 1.8 mm had the best discriminating abilities for NOCDs. The lower rate of NOCDs (8.5 vs. 37.3%, *p* < 0.001) in the group with Δcoronal MSID ≧ 1.8 mm revealed a satisfactory result in self-expanding TAVR for BAV patients. The conduction disturbance incidence in these patients was as low as or even lower than published data in tricuspid patients (1–4). It suggested that releasing prosthesis at a proper height based on MS length was an effective method to reduce the risk of NOCDs. Besides, MS guided prosthesis implantation avoided blindly higher implantation. Individualized implantation depth guided by MS not only minimized NOCDs risk but also reduce the risk of coronary occlusion or valve migration.

### Prosthesis Oversizing Ratio and Conduction Disturbances

In our study, smaller aortic root anatomy, especially the LVOT perimeter, had the best negative predictive value for NOCDs. A smaller LVOT perimeter represented higher risks of the larger oversizing ratio by LVOT perimeter, which could cause higher radial forces on the conduction system. All multivariate regression models revealed the importance of LVOT perimeter or oversizing by LVOT perimeter. Jilaihawi's study suggested that it was possible to minimize implantation depth guided by infra-annular membranous septum depth to reduce PPMI (6). Optimal implantation depth of bicuspid patients hasn't been established yet. The manufacturer recommendation of implantation depth was 3–5 mm in Evolut series self-expanding valve and 4–6 mm in Venus series self-expanding valve. With the help of cusp overlap technique, aiming for 3 mm of implantation depth in tricuspid patients can minimize the risk of conduction disturbances (37). However, the cusp overlap view can't be reached in type 0 patients and is often extreme in Type 1 L-R fusion patients. In most heavily calcified bicuspid patients, a tapered anatomy with small supra-annular structure allows the prosthesis to be deployed at a supra-annular positioning. Aiming for <3 mm based on individual MS length may be reasonable in bicuspid patients with a median infra-annular MS of 2.3 mm. However, excessively high implantation (<1 mm below annular plane) increased the risks of “Pop-out” and coronary occlusion.

In some bicuspid patients with extremely short MS, the contact of the conduction system is inevitable. Thus, we conducted a subgroup analysis of patients with infra-annular MS depth less than implantation depth. The multivariate regression model revealed that the oversizing ratio by LVOT was the only independent predictor, which could be mediated by operators. This suggested that reducing the oversizing ratio could serve as another feasible strategy to reduce conduction disturbances and avoid incomplete prosthesis expansion, annular injury or paravalvular regurgitation. BAV patients had more calcification deposition compared with tricuspid patients, which provided a supra-annular anchor position and made it possible to reduce the oversizing ratio (12). In our single center analysis, the TAVR outcome in bicuspid patients was feasible with high device success rate of 84.5% and good performance even with first generation devices. Several recent studies have also suggested the safety and effectiveness of prosthesis undersizing based on supra-annular sizing methods (8, 10, 38, 39). LIRA method, known as Level of Implantation at the RAphe (LIRA) method, was applicated in 20 raphe-type BAV patients. Undersizing prosthesis were chosen based on LIRA method, known as Level of Implantation at the Raphe method, achieved 100% device success in 20 raphe-type BAV patients. In another CASPER study (Calcium Algorithm Sizing for bicusPid Evaluation with Raphe), 70% of prosthesis were undersized according to a new algorithm and no cases of moderate or severe PVL were found (9). Now the authors are expanding the indication of CASPER algorithm in type 0 patients (NCT04817735). To sum up, reducing the oversizing ratio was a feasible strategy to reduce conduction disturbances and maintained good procedural outcome in heavily calcified bicuspid anatomy with short MS length.

### Subgroup Analysis of High-Grade Atrioventricular Block

In a subgroup analysis based on whether developed new-onset high-grade atrioventricular block, pre-existing right bundle branch block emerged as a strong predictor of new-onset high-grade atrioventricular block while coronal MS and oversizing by LVOT perimeter remained as independent predictors (Supplementary Tables 4A,B). High-grade atrioventricular block can occur when both the left bundle branch and the right bundle branch are affected. This explained the high risk of high-grade atrioventricular block and PPMI if new-onset left bundle branch block occurred in patients with pre-existing right bundle branch block. Thus, strict electrocardiography monitoring should be carried out to detect bradycardia events in this population.

### Measurement of Implantation Depth on CT or Fluoroscopy

The prosthesis implantation depth was mainly measured by fluoroscopy on NCC direction during the procedure (40, 41). However, the feasibility of this method has not been proved in BAV patients. As the coplanar view is slightly different in BAV patients. The true position of MS is between right and non-coronary leaflets in most tricuspid patients. Logically, MS measurement on fluoroscopy might be inaccurate in BAV population especially in patients under extreme projection angle in type 0 with anteroposterior cusps or Type 1 with N-L fusion. However, in our study, we found a high linear relationship between the ID measurement on CT and fluoroscopy (Supplementary Figure 1B). This suggested ID measured on fluoroscopy could also be used during procedural implantation.

## Study Limitation

The major limitation was related to the use of the first-generation device without recapturable features in the early procedure. The implantation depth was relatively lower, and MSID was numerically larger, which increased the risk of NOCDs and should be avoided in future clinical practice. However, low rates of PPMI and NOCDs in this situation highlighted the effectiveness of reducing the oversizing ratio to lower the risk of PPMI. Moreover, the study included a small population with relatively low NOCDs and PPMI rates. Thus, reported results need to be further verified in future studies. Besides, this study was unable to encompass the entire TAVR population, which made a comparison with tricuspid patients impossible.

## Conclusion

There would be more bicuspid aortic stenosis patients undergoing TAVR with the extension of indication and thus the risk of NOCDs would be highlighted for their young age compared with tricuspid aortic stenosis patients. Our study provides a practical predictive model based on MS length and LVOT perimeter. More importantly, we demonstrate the crucial role of operators and procedural strategy. It is suggested that implantation depth should be guided by MS length. Besides, reducing the oversizing ratio might be a feasible strategy to reduce conduction disturbances and maintained good procedural outcome in heavily calcified bicuspid anatomy with short MS length. Moreover, a prospective, multicenter, randomized, superiority clinical trial (NCT04722796) is ongoing to further explore the procedural strategy of BAV patients, which can verify the finding in this study. In all, appropriate individualized procedure strategy based on bicuspid aortic stenosis patients' anatomy might lead to a low incidence of NOCDs even comparable to surgery.

## Data Availability Statement

The original contributions presented in the study are included in the article/Supplementary Material, further inquiries can be directed to the corresponding authors.

## Ethics Statement

The studies involving human participants were reviewed and approved by Second Affiliated Hospital, Zhejiang University School of Medicine; Zhengzhou Cardiovascular Hospital; Fujian Medical University Union Hospital; The First Affiliated Hospital of Bengbu Medical College; Henan Provincial Chest Hospital. The patients/participants provided their written informed consent to participate in this study. Written informed consent was obtained from the individual(s) for the publication of any potentially identifiable images or data included in this article.

## Author Contributions

YG and DZ conceived and designed the study and wrote the paper. MD, YH, and JFan helped collected data and analyzed the result. SZ, JFang, SW, QH, LC, and YY provided the data and interpreted the results. HJ, XL, and JW reviewed and edited the manuscript. All authors contributed to the article and approved the submitted version.

## Funding

This paper was supported by following foundations: Natural Science Foundation of Zhejiang Province of China (LQ21H020005), Henan Province Scientific and technological project (Grant No. 192102310062), Zhengzhou Scientific and technological beneficial project (2019KJHM0006), and Anhui Provincial Key research and development project (201904a07020017).

## Conflict of Interest

HJ has been a consultant to Edwards Lifesciences and Venus Medtech and has received grant/research support from Medtronic and Abbott Vascular. The remaining authors declare that the research was conducted in the absence of any commercial or financial relationships that could be construed as a potential conflict of interest.

## Publisher's Note

All claims expressed in this article are solely those of the authors and do not necessarily represent those of their affiliated organizations, or those of the publisher, the editors and the reviewers. Any product that may be evaluated in this article, or claim that may be made by its manufacturer, is not guaranteed or endorsed by the publisher.

## Abbreviations

BAV: Bicuspid aortic valve
TAVR: Transcatheter aortic valve replacement
NOCDs: New-onset conduction disturbances
CLBBB: Complete left bundle branch block
ID: Implantation depth
MS: Membranous septum
LVOT: Left ventricular outflow tract
MSID: Membranous septum minus Implantation depth
PPMI: Permanent pacemaker implantation.
