# Supplementary material for: The Predictors of Conduction Disturbances Following Transcatheter Aortic Valve Replacement in Patients With Bicuspid Aortic Valve: A Multicenter Study
Source: Front Cardiovasc Med. 2021 Nov 29;8:757190. doi: 10.3389/fcvm.2021.757190 (PMC8667767; doi:10.3389/fcvm.2021.757190)
Supplement: Supplementary file 1 [file Data_Sheet_1.docx]

**Figure S1A Correlations between coronal MS and infra-annular MS**


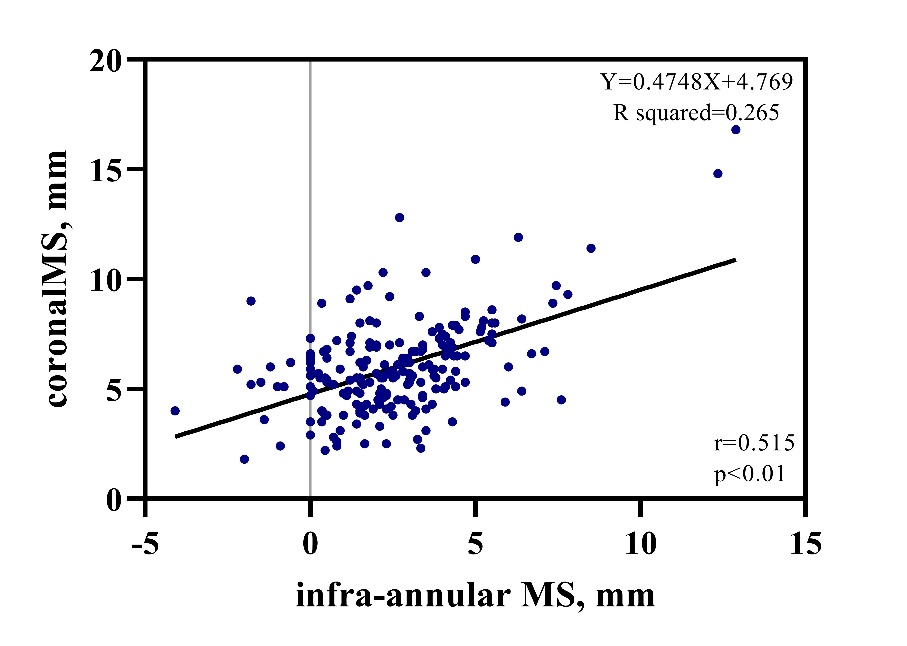


**Figure S1B Correlations between implantation depth on fluoroscopy or CT**


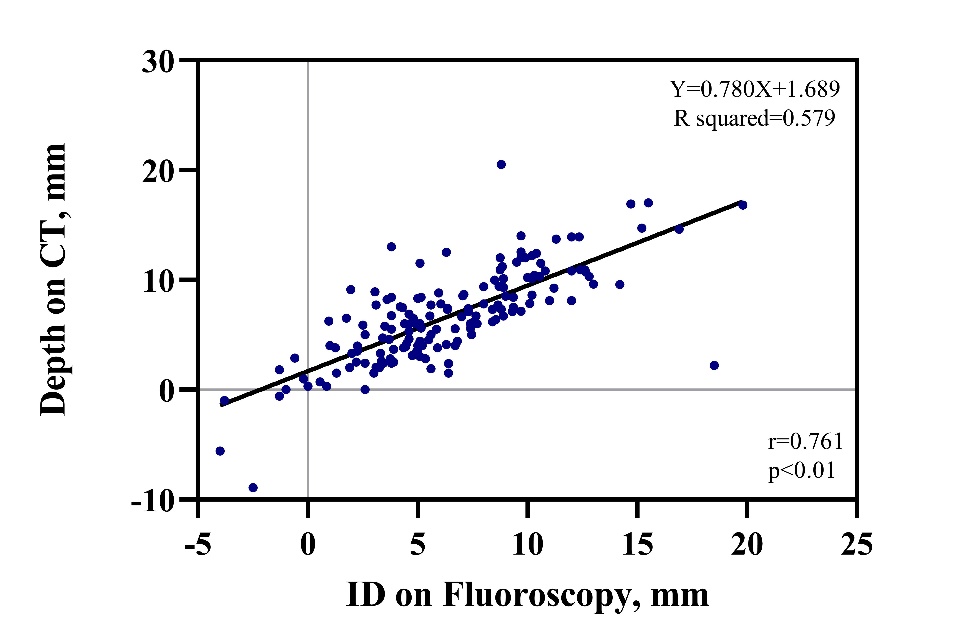


**Figure S1C Correlations between ΔMSID and Δcoronal MSID**


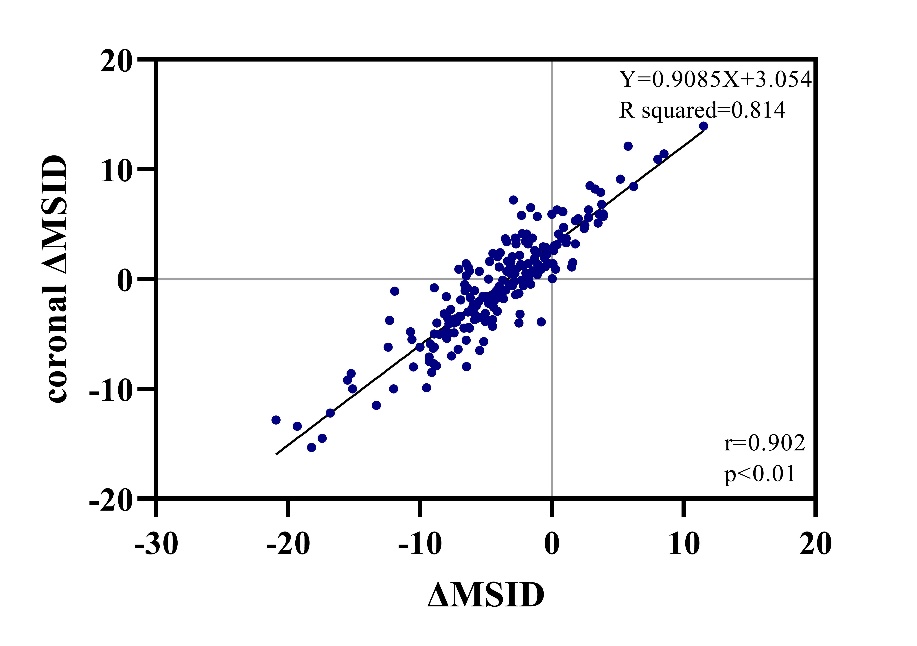


**Supplementary Table 1A**. Baseline variables comparison between different types of BAV.

|  | Total  n=209 | Type 0  n=99 | Type 1  n=79 | T-BAV  n=31 | p Value |
| --- | --- | --- | --- | --- | --- |
| Age, yrs | 75.12±6.79 | 74.1±7.02 | 76.27±6.85 | 75.45±5.43 | 0.102 |
| Male | 128(61.2%) | 54(54.5%) | 51(64.6%) | 23(74.2%) | 0.109 |
| Body mass index, kg/m^2 | 22.53±3.11 | 22.31±2.98 | 22.79±3.28 | 22.62±3.07 | 0.589 |
| Diabetes Mellitus | 41(19.6%) | 21(21.2%) | 14(17.7%) | 6(19.4%) | 0.843 |
| Hypertension | 100(47.8%) | 49(49.5%) | 36(45.6%) | 15(48.4%) | 0.871 |
| COPD | 43(20.6%) | 21(21.2%) | 14(17.7%) | 8(25.8%) | 0.626 |
| CKD stage IV/V | 4(1.9%) | 1(1.0%) | 3(3.8%) | 0(0.0%) | 0.370 |
| STS score, % | 6.80±4.51 | 6.10±3.77 | 7.77±5.41 | 6.54±3.79 | **0.045** |
| Atrial Fibrillation/Flutter | 32(15.3%) | 14(14.1%) | 12(15.2%) | 6(19.4%) | 0.781 |
| Preexisting LBBB | 18(8.6%) | 11(11.1%) | 4(5.1%) | 3(9.7%) | 0.344 |
| Preexisting RBBB | 17(8.1%) | 9(9.1%) | 7(8.9%) | 1(3.2%) | 0.654 |
| Baseline echocardiographic variables | | | | | |
| Mean gradient, mmHg | 60.25±22.92 | 60.63±23.63 | 60.77±22.59 | 57.74±21.97 | 0.804 |
| AVA, cm^2 | 0.51±0.29 | 0.53±0.26 | 0.47±0.33 | 0.59±0.31 | 0.133 |
| Max velocity, m/s | 4.9±0.96 | 4.99±0.94 | 4.80±1.03 | 4.84±0.83 | 0.388 |
| LVEF, % | 53.81±13.21 | 54.49±14.07 | 52.36±12.32 | 55.34±12.65 | 0.444 |
| Computed tomography variables | | | | | |
| Annulus Area, mm^2 | 477.22±106.73 | 459.53±100.09 | 491.67±117.29 | 496.87±92.23 | 0.073 |
| Annulus perimeter | 78.66±8.35 | 77.25±7.97 | 79.69±8.96 | 80.56±7.33 | 0.059 |
| Annular eccentricity index | 0.23±0.07 | 0.23±0.08 | 0.24±0.06 | 0.24±0.06 | 0.825 |
| LVOT area, mm^2 | 508.29±154.72 | 499.54±153.44 | 509.46±163.25 | 533.26±137.37 | 0.571 |
| LVOT perimeter | 84.52±13.23 | 83.85±12.78 | 84.86±14.64 | 85.79±10.92 | 0.746 |
| LVOT eccentricity index * | 0.31±0.08 | 0.33±0.09 | 0.30±0.07 | 0.29±0.07 | **0.013** |
| LVOT calcification | 36(17.2%) | 15(15.2%) | 13(16.5%) | 8(25.8%) | 0.381 |
| AA diameter at 40mm, mm | 38.85±3.88 | 39.58±3.89 | 38.65±3.86 | 36.98±3.29 | **0.004** |
| Max AA diameter, mm | 42.62±4.68 | 43.87±4.57 | 42.09±4.63 | 39.97±3.81 | **<0.001** |
| RCA height, mm | 17.34±3.85 | 17.98±4.44 | 16.42±2.98 | 17.66±3.40 | **0.023** |
| LCA height, mm | 16.01±4.09 | 17.4±4.21 | 14.74±3.79 | 14.8±2.91 | **<0.001** |
| Aortic root angulation | 52.82±10.49 | 54.15±10.53 | 52.57±10.13 | 49.19±10.66 | 0.068 |
| Infra-annular MS length, mm | 2.56±2.27 | 2.48±2.22 | 2.44±2.21 | 3.09±2.57 | 0.364 |
| Coronal MS length, mm | 5.98±2.09 | 5.58±1.92 | 6.31±2.25 | 6.44±2.04 | **0.030** |

Values are presented as mean ± SD or n (%). p Values in bold are statistically significant, which is calculated by ANOVA (Analysis of Variance).

AA = ascending aorta; AVA = aortic valve orifice area; BAV = bicuspid aortic valve; COPD = chronic obstructive pulmonary disease; CKD = chronic kidney disease; F-BAV = functional bicuspid aortic valve; LBBB = left bundle branch block; LCA = left coronary height; LVEF = left ventricular ejection fraction; LVOT = left ventricular outflow tract; MS = membranous septum; RBBB = right bundle branch block; RCA = right coronary artery; STS = Society of Thoracic Surgeons

|  | Total  n=209 | Type 0  n=99 | Type 1  n=79 | T-BAV  n=31 | p Value |
| --- | --- | --- | --- | --- | --- |
| Pre-dilatation | 204(97.6%) | 99(100%) | 75(94.9%) | 30(96.8%) | 0.058 |
| Post-dilatation | 150(71.8%) | 71(71.7%) | 55(69.6%) | 24(77.4%) | 0.716 |
| Second valve implantation | 17(8.1%) | 7(7.1%) | 4(5.1%) | 6(19.4%) | 0.057 |
| Oversizing by annulus perimeter, % | 4.9±8.7 | 5.6±8.4 | 4.6±8.5 | 3.5±9.9 | 0.431 |
| Oversizing by annulus area, % | 15.8±19.4 | 17.5±19.1 | 14.7±18.8 | 13.2±22 | 0.460 |
| Oversizing by LVOT perimeter, % | -1.2±12.9 | -1.6±12.7 | -0.4±13.6 | -2.2±12 | 0.772 |
| Oversizing by LVOT area, % | 13.4±31.7 | 12.6±28.8 | 16.4±36.4 | 8.7±27.4 | 0.488 |
| Implantation depth, mm | 6.63±4.26 | 5.75±3.85 | 7.03±3.95 | 8.44±5.51 | **0.005** |
| ΔMSID, mm | -4.07±4.85 | -3.26±4.43 | -4.59±4.31 | -5.35±6.79 | 0.055 |
| Implant depth > infra-annular MS length | 173(82.8%) | 79(79.8%) | 68(86.1%) | 26(83.9%) | 0.537 |
| Δcoronal MSID, mm | -0.86±4.85 | -0.52±4.88 | -0.80±4.43 | -2.03±5.68 | 0.322 |
| New-onset conduction disturbances | 61(29.2%) | 29(29.3%) | 21(26.6%) | 11(35.5%) | 0.652 |
| Post New LBBB | 42(20.1%) | 24(24.2%) | 12(15.2%) | 6(19.4%) | 0.324 |
| Post New HAVB | 21(10%) | 6(6.1%) | 10(12.7%) | 5(16.1%) | 0.146 |
| Post PPMI | 17(8.1%) | 4(4%) | 8(10.1%) | 5(16.1%) | 0.061 |

**Supplementary Table 1B**. Procedural Characteristics and conduction abnormalities

Values are presented as mean ± SD or n (%). p Values in bold are statistically significant, which is calculated by ANOVA (Analysis of Variance).

T-BAV = tricommissural raphe-type bicuspid aortic valve; HAVB = high-grade atrioventricular block; LBBB = left bundle branch block; LVOT = left ventricular outflow tract; MS = membranous septum; ΔMSID = membranous length minus implantation depth; NOCDs = New-Onset Conduction Disturbances; PPMI = permanent pacemaker implantation; RBBB = right bundle branch block.

**Supplementary Table 1C**. Detail Comparison between different types of BAV

|  | Total  n=209 | Type 0  n=99 | Type 1  n=79 | T-BAV  n=31 | p Value | Type 0 vs. Type 1 | Type 0 vs. T-BAV | Type 1 vs. T-BAV |
| --- | --- | --- | --- | --- | --- | --- | --- | --- |
| STS score, % | 6.80±4.51 | 6.10±3.77 | 7.77±5.41 | 6.54±3.79 | **0.045** | 0.062 | 0.919 | 0.450 |
| LVOT eccentricity index | 0.31±0.08 | 0.33±0.09 | 0.30±0.07 | 0.29±0.07 | **0.013** | **0.037** | **0.037** | 0.916 |
| AA diameter at 40mm, mm | 38.85±3.88 | 39.58±3.89 | 38.65±3.86 | 36.98±3.29 | **0.004** | 0.105 | **0.001** | **0.039** |
| Max AA diameter, mm | 42.62±4.68 | 43.87±4.57 | 42.09±4.63 | 39.97±3.81 | **<0.001** | **0.009** | **<0.001** | **0.028** |
| RCA height | 17.34±3.85 | 17.98±4.44 | 16.42±2.98 | 17.66±3.40 | **0.023** | **0.007** | 0.688 | 0.122 |
| LCA height | 16.01±4.09 | 17.4±4.21 | 14.74±3.79 | 14.8±2.91 | **<0.001** | **<0.001** | **0.001** | 0.943 |
| Coronal MS length, mm | 5.98±2.09 | 5.58±1.92 | 6.31±2.25 | 6.44±2.04 | **0.030** | **0.022** | **0.046** | 0.761 |
| Implantation Depth | 6.63±4.26 | 5.75±3.85 | 7.03±3.95 | 8.44±5.51 | **0.005** | 0.091 | **0.046** | 0.485 |

One-way analysis of variance (ANOVA) with LSD or Dunnett multiple comparison test were performed. Values are presented as mean ± SD or n (%). p Values in bold are statistically significant.

AA = ascending aorta; T-BAV = tricommissural raphe-type bicuspid aortic valve; LCA = left coronary height; LVOT = left ventricular outflow tract; MS = membranous septum; RCA = right coronary artery; STS = Society of Thoracic Surgeons

**Supplementary Table 2.** Detail pre- and post-operative arrhythmic characteristics of Patients with New-onset HAVB

|  | Preoperative Arrhythmia | Postoperative Arrhythmia | PPMI & time |
| --- | --- | --- | --- |
| Pt 1-7 | no conduction disturbance | 3°AVB | PPMI before discharge |
| Pt 8 | no conduction disturbance | Adams-Stroke syndrome, syncope, HAVB (P waves do not conduct to the ventricles for 50 seconds) | PPMI before discharge |
| Pt 9 | no conduction disturbance | HAVB (transient 3°AVB, 2°AVB type 2 (Mobitz II)) | patient and his family refuse PPMI |
| Pt 10 | 1°AVB | Frequent 3°AVB, sinus arrest for 4.72 seconds, CRBBB | PPMI before discharge |
| Pt 11 | 1°AVB | HAVB (transient 3°AVB), CLBBB, 1°AVB, 2°AVB type 1; no 3°AVB according to Holter and electrocardiography before discharge. | patient and his family refuse PPMI |
| Pt 12 | Afib | 2°AVB type 2 (Mobitz II) | PPMI before discharge |
| Pt 13 | Afib | Frequent 3°AVB, 2°AVB type 2 (Mobitz II) | PPMI before discharge |
| Pt 14 | Afib, CRBBB | Frequent 3°AVB, CLBBB, CRBBB | PPMI before discharge |
| Pt 15 | Afib, CRBBB | Transient HAVB, CRBBB before discharge. HAVB in 10 days electrocardiography | PPMI 10 days after discharge |
| Pt 16 | Afib, LBBB (undefined) | Afib with Long RR intervals | PPMI before discharge |
| Pt 17-18 | CRBBB | 3°AVB | PPMI before discharge |
| Pt 19 | CRBBB | HAVB (transient 3°AVB) and CRBBB , no 3°AVB according to Holter before discharge. | CRBBB at discharge, no PPMI |
| Pt 20 | CRBBB | Transient HAVB, CRBBB, LAH, no 3°AVB according to Holter before discharge. | no PPMI |
| Pt 21 | CRBBB, 1°AVB | 3°AVB, persistent pacemaker heart rate | PPMI before discharge |

Afib: atrial fibrillation; AVB: atrioventricular block; CRBBB: complete right bundle branch block; LBBB: left bundle branch block; LAH: left anterior hemiblock; LPH: left posterior hemiblock; 1°AVB: first-degree atrioventricular block; 2°AVB: second-degree atrioventricular block; 3°AVB: third-degree atrioventricular block; PPMI: permanent pacemaker implantation;

**Supplementary Table 3. Discriminatory ability of models to New-Onset Conduction Disturbances after TAVR in BAV patients.**

|  | C-statistic* |
| --- | --- |
| Age >73yrs | 0.592(0.509-0.675) |
| LVOT perimeter <66.8 mm | 0.557(0.468-0.646) |
| Infra-annular MS <3.7 mm | 0.572(0.489-0.655) |
| Coronal MS <4.9mm | 0.621(0.535-0.708) |
| ΔMSID <-2.9mm | 0.632(0.551-0.712) |
| Δcoronal MSID <1.8mm | 0.641(0.565-0.718) |
| Oversizing by LVOT perimeter >3.2% | 0.633(0.549-0.718) |
| Model1: Age >73 yrs+ LVOT perimeter <66.8mm+ Infra-annular MS <3.7mm | 0.672(0.591-0.752) |
| Model2: Age >73 yrs+ LVOT perimeter <66.8mm+ Coronal MS <4.9mm | 0.692(0.609-0.774) |
| Model3: Age >73 yrs+ ΔMSID <-2.9mm+ Oversizing by LVOT perimeter >3.2% | 0.752(0.679-0.824) |
| Model4: Age >73 yrs+ Δcoronal MSID<1.8mm+ Oversizing by LVOT perimeter >3.2% | 0.768(0.699-0.837) |

* Concordance-statistic (c-statistic) was evaluated using the area under the receiver operating characteristic (ROC) curve (AUC).

Comparison of AUCs between Infra-annular MS and Coronal MS, ΔMSID, Δcoronal MSID was significant (P = 0.005, 0.026, 0.008, respectively). Comparison of AUCs between four multivariate models was non-significant except Model1 vs Model4 (P = 0.027).

LVOT = left ventricular outflow tract; MS = membranous septum; ΔMSID = membranous length minus implantation depth;

| **Supplementary Table 4A**. Bicuspid aortic stenosis patients’ characteristics and HAVB | | | | |
| --- | --- | --- | --- | --- |
|  | Total  (n=209) | No HAVB  (n=188) | HAVB  (n=21) | p Value |
| Age, yrs | 75.12±6.79 | 74.84±6.69 | 77.62±7.34 | 0.075 |
| Male | 128(61.2%) | 116(61.7%) | 12(57.1%) | 0.684 |
| Body mass index, kg/m^2 | 22.53±3.11 | 22.55±3.09 | 22.39±3.35 | 0.818 |
| Diabetes Mellitus | 41(19.6%) | 40(21.3%) | 1(4.8%) | 0.129 |
| Hypertension | 100(47.8%) | 89(47.3%) | 11(52.4%) | 0.661 |
| COPD | 43(20.6%) | 40(21.3%) | 3(14.3%) | 0.640 |
| CKD Stage 4-5 | 4(1.9%) | 4(2.1%) | 0(0.0%) | 1.000 |
| STS score, % | 5.487(3.626,9.052) | 5.482(3.816,8.925) | 5.6(3.180,10.009) | 0.872 |
| Atrial Fibrillation/Flutter | 32(15.3%) | 27(14.4%) | 5(23.8%) | 0.412 |
| Preexisting LBBB | 18(8.6%) | 18(9.6%) | 0(0.0%) | 0.283 |
| Preexisting RBBB | 17(8.1%) | 10(5.3%) | 7(33.3%) | **<0.001** |
| Mean gradient, mmHg | 56.0(43.0,70.5) | 56.0(43.0,71.0) | 56.0(50.0,69.0) | 0.559 |
| Max velocity, m/s | 4.9(4.2,5.5) | 4.9(4.2,5.5) | 5(4.4,5.3) | 0.721 |
| LVEF, % | 57.0(46.0,63.4) | 56.9(44.0,63.4) | 57.5(52.3,63.7) | 0.499 |
| Type of BAV |  |  |  | 0.165 |
| 0 | 99(47.4%) | 93(49.5%) | 6(28.6%) |  |
| 1 | 79(37.8%) | 69(36.7%) | 10(47.6%) |  |
| F-BAV | 31(14.8%) | 26(13.8%) | 5(23.8%) |  |
| Annulus perimeter, mm | 77.2(73.2,82.9) | 77.2(73.0,83.6) | 78.1(74.4,82.3) | 0.885 |
| LVOT perimeter, mm | 83.8(75.4,92.7) | 83.9(75.5,93.2) | 80.9(74.3,91.3) | 0.341 |
| Aortic root angulation | 52.82±10.49 | 52.69±10.49 | 54.00±10.66 | 0.587 |
| Infra-annular MS length, mm | 2.3(1.2,3.9) | 2.3(1.2,4.0) | 2.1(1.5,3.5) | 0.642 |
| Coronal MS length, mm | 5.7(4.7,7.0) | 5.9(4.8,7.1) | 4.8(4.1,5.5) | **0.003** |
| Oversizing by annulus perimeter, % | 4.90± 8.70 | 4.62±8.82 | 7.42±7.02 | 0.162 |
| Oversizing by annulus area, % | 15.80±19.40 | 15.25±19.71 | 20.59±16.32 | 0.233 |
| Oversizing by LVOT perimeter, % | -1.20±12.90 | -1.89±12.82 | 4.59±12.89 | **0.029** |
| Oversizing by LVOT area, % | 9.1(-7.8,29.6) | 8.2(-7.9,29) | 18.3(2.1,36.7) | 0.108 |
| Pre-dilatation | 204(97.6%) | 185(98.4%) | 19(90.5%) | 0.080 |
| Post-dilatation | 150(71.8%) | 133(70.7%) | 17(81%) | 0.324 |
| Second valve implantation | 17(8.1%) | 16(8.5%) | 1(4.8%) | 0.861 |
| Implant depth, mm | 6.3(3.9,9.0) | 6.3(3.8,9.2) | 6.3(4.6,7.3) | 0.732 |
| ΔMSID, mm | -4(-6.6,-1.3) | -4.1(-6.8,-1.1) | -3.4(-5.8,-1.8) | 0.845 |
| Δcoronal MSID, mm | -0.86±4.85 | -0.85±5.05 | -0.89±2.49 | 0.958 |

Values are presented as mean±SD or median (Quartile1,Quartile3) or n (%). p Values in bold are statistically significant.

CKD = chronic kidney disease; COPD = chronic obstructive pulmonary disease; F-BAV = functional bicuspid aortic valve; HAVB = high-grade atrioventricular block; STS = Society of Thoracic Surgeons; LBBB = left bundle branch block; LVEF = left ventricular ejection fraction; LVOT = left ventricular outflow tract; RBBB = right bundle branch block; MS = membranous septum; ΔMSID = infra-annular MS length minus implantation depth; Δcoronal MSID = coronal MS length minus implantation depth.

**Supplementary table 4B.** Logistic regression for the predictors of high-grade atrioventricular block

|  | Univariate analysis | | Multivariate analysis model 01* | | Multivariate regression 02† | |
| --- | --- | --- | --- | --- | --- | --- |
|  | p Value | OR(95%CI) | p Value | OR(95%CI) | p Value | OR(95%CI) |
| Pre-existing RBBB | <0.001 | 8.90 (2.94-26.97) | 0.001 | 8.36(2.50-27.89) | <0.001 | 10.48(3.06-35.92) |
| Coronal MS length <5.5 mm | 0.002 | 5.86(1.90-18.10) | 0.004 | 5.78(1.75-19.12) | - | - |
| Pre-dilatation | 0.018 | 0.14(0.03-0.71) | - | - | - | - |
| Δcoronal MSID <1.8 mm | 0.035 | 8.92(1.17-68.08) | - | - | 0.030 | 10.76(1.26-91.57) |
| Oversizing by LVOT perimeter >6.4% | 0.008 | 3.50(1.39-8.77) | 0.010 | 3.80(1.38-10.50) | 0.011 | 3.68(1.34-10.11) |

Multivariate logistic regression included parameters with a p value < 0.05 without significant multicollinearity using forward Likelihood Ratio method.

* c-statistics: 0.805 (95%CI: 0.699-0.911), p <0.001

† c-statistics: 0.791 (95%CI: 0.688-0.895), p <0.001

LVOT= left ventricular outflow tract; MS= membranous septum; RBBB= right bundle branch block; Δcoronal MSID=coronal MS length minus implantation depth

**Supplementary Table 5A.** Predictors of NOCDs in ID ≥ Infra-annular MS group

|  | MS*≤ID  (n=173) | No NOCDs  (n=116) | New NOCDs  (n=57) | p Value |
| --- | --- | --- | --- | --- |
| Age, yrs | 74.87±6.87 | 73.97±6.99 | 76.72±6.35 | **0.013** |
| Diabetes Mellitus | 31(17.9%) | 16(13.8%) | 15(26.3%) | **0.044** |
| MS length*, mm | 2.1(0.8, 3.4) | 2.1(0.5, 3.3) | 2.3(1.5, 3.5) | 0.217 |
| MS length* <5 | 162(93.6%) | 108(93.1%) | 54(94.7%) | 1.000 |
| Pre-dilatation | 169(97.7%) | 113(97.4%) | 56(98.2%) | 1.000 |
| Post-dilatation | 126(72.8%) | 83(71.6%) | 43(75.4%) | 0.589 |
| Second valve implantation | 15(8.7%) | 8(6.9%) | 7(12.3%) | 0.258 |
| Annular area | 461.5(410.7, 528.1) | 470.8(421.1, 556.0) | 433.7(403.5, 509.4) | **0.030** |
| Annular perimeter | 77.6(73.4, 83.2) | 78.8(74.3, 85.2) | 75.4(72.2, 81.2) | **0.025** |
| LVOT area | 488.8(406.9, 609.9) | 494.4(413.6, 630.1) | 453.8(380.9, 564.7) | **0.017** |
| LVOT perimeter | 83.8(75.5, 93.2) | 85.4(76.7, 94.8) | 80.9(73.7, 88.1) | **0.012** |
| Oversizing by annular perimeter | 4.7±8.9 | 3.1±9.5 | 7.9±6.7 | **<0.001** |
| Oversizing by LVOT perimeter | -1.3±13.2 | -3.8±12.9 | 3.8±12.2 | **<0.001** |
| Implantation Depth | 7.3(4.8, 9.7) | 6.8(4.5, 9.5) | 7.7(5.9, 10.4) | 0.083 |
| △MSID | -4.9(-7.4, -2.7) | -4.4(-7.4, -2.4) | -5.4(-7.4, -3.5) | 0.203 |

Values are presented as mean±SD or median (Quartile1,Quartile3) or n (%). p Values in bold are statistically significant.

MS* = Infra-annular membranous septum; LVOT = left ventricular outflow tract; NOCDs = New-Onset Conduction Disturbances; ΔMSID = infra-annular MS length minus implantation depth.

**Supplementary Table 5B.** Logistic regression of NOCDs in ID ≥ Infra-annular MS group

|  | Univariate regression | | Multivariate regression | |
| --- | --- | --- | --- | --- |
|  | p Value | OR(95%) | p Value | OR(95%) |
| Age, per 1 yrs | **0.014** | 1.06 (1.01-1.12) | **0.020** | 1.07 (1.01-1.12) |
| Diabetes Mellitus | **0.047** | 2.23 (1.01-4.92) | - | - |
| Infra-annular MS length, per 1mm | 0.415 | 1.07 (0.91-1.24) | - | - |
| Implantation Depth, per 1mm | 0.214 | 1.05(0.97-1.15) | - | - |
| △MSID, per 1mm | 0.968 | 0.97 (0.89-1.05) | - | - |
| Oversizing by LVOT perimeter, per 1% | **0.001** | 1.05 (1.02-1.08) | **0.001** | 1.05 (1.02-1.08) |

Forward LR, univariate ≤ 0.05.

Multivariate logistic regression included parameters with a p value < 0.05 without significant multicollinearity using forward Likelihood Ratio method.

MS* = Infra-annular membranous septum; LVOT = left ventricular outflow tract; NOCDs = New-Onset Conduction Disturbances; ΔMSID = infra-annular MS length minus implantation depth.
